# Nonalcoholic Fatty Liver Disease Risk and Proprotein Convertase Subtilisin Kexin 9 in Familial Hypercholesterolemia Under Statin Treatment

**DOI:** 10.3390/nu16213686

**Published:** 2024-10-29

**Authors:** Masato Hamasaki, Naoki Sakane, Kazuhiko Kotani

**Affiliations:** 1 Division of Community and Family Medicine, Jichi Medical University, 3311-1 Yakushiji, Shimotsuke 329-0498, Japan; masatohamasaki@jichi.ac.jp; 2 Division of Preventive Medicine, Clinical Research Institute, National Hospital Organization Kyoto Medical Center, 1-1 Fushimi-Ku, Kyoto 612-8555, Japan; nsakane@gf6.so-net.ne.jp

**Keywords:** fatty acids, fatty liver disease, HMG-CoA reductase inhibitor, lipid-lowering therapy

## Abstract

Background/Objectives: Fatty acids are involved in some hepatic disorders. The proprotein convertase subtilisin kexin 9 (PCSK9) inhibits the uptake of low-density lipoproteins (LDLs), which contain lipids, into the liver and may thus be associated with nonalcoholic fatty liver disease (NAFLD), a cardiovascular disorder (CVD) risk. Statins reduce blood LDL–cholesterol (LDL-C) levels and CVD risk and can attenuate the development of NAFLD while increasing blood PCSK9 levels. Methods: We investigated the correlation between PCSK9 and liver conditions in patients with familial hypercholesterolemia (FH), a CVD risk population with elevated blood LDL-C levels, under statin treatment. Blood tests for lipids, PCSK9, and liver function (aspartate aminotransferase [AST] and alanine aminotransferase [ALT]) were performed in patients with FH taking statins (*n* = 25, mean age = 57 years, 12% of males). The ALT:AST ratio was used as a marker of NAFLD risk. Results: The mean LDL-C level was 3.38 mmol/L, and the median PCSK9 level was 312 ng/mL. The median ALT:AST ratio was 0.88. A significant negative correlation was observed between the PCSK9 and ALT:AST ratio (β = −0.67, *p* < 0.05). Conclusions: Their negative correlation might give a hypothetical insight into the effect of statin treatment on the development of NAFLD, in relation to PCSK9 behavior, in patients with FH.

## 1. Introduction

Fatty acids are lipids that are mainly generated in the liver and included in various lipoprotein species [1]. Fatty acids are involved in the pathophysiology of nonalcoholic fatty liver disease (NAFLD), a lipid accumulation disorder in the liver [2]. NAFLD includes fatty liver and steatohepatitis, which is commonly observed in ~25% of the general population [2]. Hyperlipidemias, such as hypertriglyceridemia and hypercholesterolemia, are associated with the development of NAFLD [3]. Typically, NAFLD progress to severe liver fibrosis and is a risk factor for cardiovascular disease (CVD) [3,4]. The prevention of CVD by NAFLD risk management is necessary.

Familial hypercholesterolemia (FH) is a disorder characterized genetically by elevated blood low-density lipoprotein (LDL) cholesterol (LDL-C) levels, a risk factor for CVD [5,6,7,8], and the proprotein convertase subtilisin kexin 9 (PCSK9) is one of the key molecules in the pathophysiology of FH [9]. PCSK9 inhibits the uptake of LDL via LDL receptors in the liver, thereby elevating blood LDL levels [9,10]. Furthermore, a genetic variant of the gain-of-function of PCSK9 inhibits LDL uptake via LDL receptors, which is one of the causative genes of FH [11,12]. Thus, there is a positive correlation between LDL-C and PCSK9 levels in the blood [13], and the high PCSK9 level is also a risk factor for CVD [14].

Statins, which are 3-hydroxy-3-methylglutaryl-CoA inhibitors, are lipid-lowering drugs [15] that inhibit the cholesterol generation pathway, resulting in a reduction in the total amount of cholesterol in the liver and LDL levels in the blood [6]. Statins are used to reduce CVD risk in patients with FH [15]. In contrast, statins are known to upregulate PCSK9 levels in the liver [16].

Of interest, PCSK9 levels are also elevated in patients with NAFLD [17]. A positive correlation has been reported between PCSK9 and NAFLD risk in the absence of statin treatment [17]. Statins can prevent the development of NAFLD and reduce CVD risk in patients with NAFLD [6,18,19]. In this case [6,18,19], although PCSK9 levels were high in patients with NAFLD under statin treatment, we wondered why the treatment could reduce the development of NAFLD and CVD risk. Anti-inflammatory and anti-oxidative effects of statins have been assumed for the observation [6,18,19]. The examination of correlation between PCSK9 and liver function may further elucidate the mechanisms underlying the linkage between hepatic disorders and CVD risk reduction following statin treatment.

Therefore, in this study, we aimed to investigate the correlation between PCSK9 and liver function in patients with FH undergoing statin treatment. Here, the aminotransferase (ALT)/aspartate aminotransferase (AST) ratio was used as a marker of liver function for NAFLD risk because an earlier study reported a positive association between the ALT:AST ratio (even at its normal level) and NAFLD risk [20,21].

## 2. Materials and Methods

This cross-sectional study included 25 patients (mean age = 57 years old, 12% males) with FH who were being treated with the maximum dose of statins, such as atorvastatin (40 mg/day), pitavastatin (4 mg/day), and simvastatin (20 mg/day). Two patients received statins plus ezetimibe, a Niemann–Pick C1-like 1 inhibitor (10 mg/day). The clinical diagnosis of FH was based on standard criteria in Japan (i.e., elevated LDL-C level, xanthomas, family history of FH or premature coronary artery disease) [6]. Patients with apparent CVD, acute conditions (i.e., infectious diseases), muscle pain/weakness by statin treatment, and hepatic disorders due to viral infections and alcoholic consumption were excluded. This study was conducted following the Declaration of Helsinki and approved by the Ethics Review Committee of Jichi Medical University. Written informed consent was obtained from all the patients.

Body mass index (BMI) was calculated as weight in kilograms divided by the square of height in meters. Serum levels of total cholesterol and high-density lipoprotein (HDL) cholesterol (HDL-C) were determined using enzymatic methods, with an intra- and inter-assay coefficient of variation (CV) of 0.4% for total cholesterol and an intra-assay CV of 1.1% and an inter-assay CV of 2.4% for HDL-C, respectively (Sekisui Medical Co., Ltd., Tokyo, Japan) [22,23]. Serum triglyceride levels were measured using an enzymatic method with an intra- and inter-assay CV of 0.5% and 0.4%, respectively (Sekisui Medical Co., Ltd., Tokyo, Japan). Serum LDL-C levels were calculated using the Friedewald equation, given that all patients had triglycerides < 4.5 mmol/L [6]. Serum PCSK9 levels were measured using the enzyme-linked immunosorbent assay with an intra- and inter-assay CV ≤ 7.0% (R&D Systems, Minneapolis, MN, USA). As an increase in the ALT:AST ratio is indicative of potential NAFLD [20,21], serum ALT and AST levels were both measured using an enzymatic method with an intra- and inter-assay CV within 2.5% and 5.0% [24].

Three major gene variants (LDLR, PCSK9, and APOB) of FH were examined using next-generation sequencing with 50 ng of genomic DNA via NextSeq500 (Illumina, San Diego, CA, USA). The detected variants of amino acid substitutions or splice regions were interpreted using the ClinVar database [25].

Data are expressed as the mean ± standard deviation and median (interquartile range) for parametric and nonparametric variables, respectively, and as numbers (%) for categorical variables. Pearson’s correlation test and multiple linear regression model adjusted for LDL-C were used to analyze the correlation between PCSK9 and the ALT:AST ratio. Owing to the skewed distribution, the PCSK9 levels and ALT:AST ratio were log-transformed when applying Pearson’s correlation test. Spearman’s rank correlation test was also used for the skewed-distributed variables without log transformation. The threshold for statistical significance was set at *p* < 0.05. All statistical procedures were performed using R statistical software version 4.3.2. (https://www.R-project.org/ [accessed on 24 October 2024]).

## 3. Results

The clinical characteristics of patients with FH are listed in Table 1. There were no patients with diabetes mellitus. The mean LDL-C level was 3.38 mmol/L, and the median PCSK9 level was 312 ng/mL. The mean ALT level was 21.7 U/L, and the mean AST level was 23.1 U/L. The median ALT:AST ratio was 0.88.

**Figure 1:**
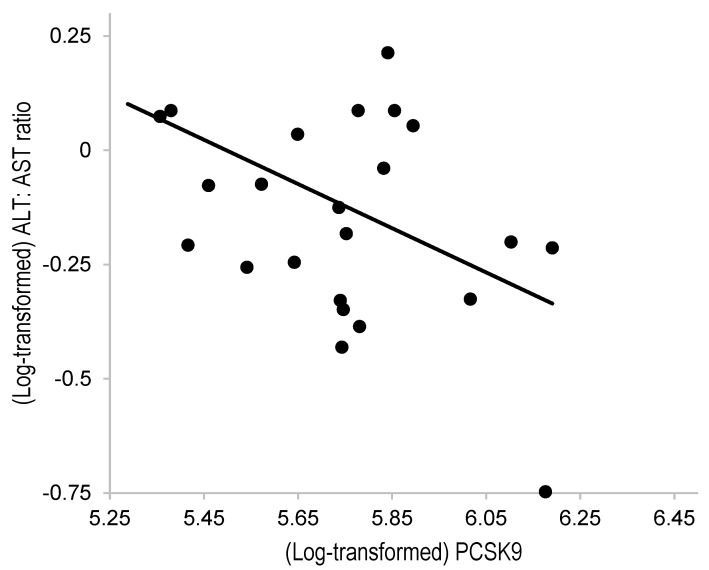
Correlation between the PCSK9 and ALT:AST ratio in patients with FH under statin treatment. ALT, alanine aminotransferase; AST, aspartate aminotransferase; PCSK9, proprotein convertase subtilisin/kexin type 9. The PCSK9 levels and ALT:AST ratio were log-transformed. Pearson’s correlation coefficient (*r*) was = −0.45 (*p* = 0.02).

Table 2 shows the correlation between PCSK9 and each variable in Pearson’s correlation test. Although PCSK9 showed no significant correlation with most variables, there was a negative correlation between the PCSK9 and LDL-C levels (*r* = −0.45, *p* = 0.02 [*rho* = −0.29 in Spearman’s rank correlation test], Figure 1). Further, the multiple linear regression model revealed a significant negative correlation between the PCSK9 levels and ALT:AST ratio after adjusting for LDL-C levels (*β* = −0.67, Table 2).

Genetic tests showed that one patient possessed the *PCSK9* p.E32K variant and two patients possessed the *LDLR* p.C325Y and p.C338S variants. The PCSK9 levels in these patients were 212, 312, and 217 ng/mL, respectively. The effect of the variants on PCSK9 levels in the correlation test was not evident in this population.

## 4. Discussion

In this study, there was a significant negative correlation between the PCSK9 levels and ALT:AST ratio, a marker of NAFLD risk, in patients with FH “under” statin treatment. Although a positive correlation was reported in the “absence” of statin treatment [17], this study found a different correlation “under” statin treatment. This correlation may provide an idea of the effect of statin treatment on the modification of the pathophysiology of NAFLD and CVD risk in hypercholesterolemic states [6,18,19].

The development of a precise serum marker for NAFLD remains an issue [20], so this study used the AST:ALT ratio. This ratio is thought to be suitable for observing NAFLD risk among non-obese patients, while ALT alone is used for observing NAFLD risk among obese patients [20,21]. In this study population, the mean BMI (23.2 kg/m^2^, Table 1) showed a non-obese level [26]. The use of the ALT:AST ratio as an NAFLD-related marker was thought to be reasonable.

Two possible explanations were raised for the negative correlation between the PCSK9 levels and ALT:AST ratio. First, statin treatment can attenuate NAFLD via a reduction in lipid accumulation in the liver [19,27,28], while statin treatment can upregulate PCSK9 expression in the liver and PCSK9 levels in the blood [13,16,29,30]. Namely, in a usual situation, LDL is internalized by LDL receptors; then, LDL is transported through endosome in a clathrin-mediated manner and degraded at lysosome in the liver [9]. The accumulation of cholesterol ester and fatty acids catabolized from LDL forms lipid droplets and induces endoplasmic reticulum stress, which leads to liver damage as hepatic inflammation and NAFLD [31,32]. Under statin treatment, those drugs inhibit the cholesterol generation pathway [6] and reduce cholesterol accumulation in the liver, which leads to mitigating hepatic inflammation and attenuating NAFLD [27,28]. Simultaneously, the inhibition of statins on cholesterol generation activates sterol regulatory element-binding protein 2 (a molecule for cholesterol biosynthesis), and this molecule binds to the SRE motif of the PCSK9 gene, which upregulates PCSK9 expression in the liver and secretion in the blood [29]. Thus, a negative correlation would appear between the PCSK9 levels and ALT:AST ratio, a marker of NAFLD risk, as observed in this study. The greater the rate of LDL-C reduction with statin treatment, the greater the expression of PCSK9 and its blood levels [16]. Because patients with FH generally exhibit high LDL-C levels, and then the maximum dose of statins is administered to stably reduce the LDL-C levels, this negative correlation may have more clearly appeared.

Another possible explanation is the assumption that PCSK9 could have a direct preventive effect against NAFLD. Of note, there is a report that PCSK9 inhibits lipid uptake and mitigates lipid accumulation in the liver [33]. PCSK9-knockout cells are also reported to have increased lipid droplets in vivo [34]. Further studies are thus required to elucidate the mechanisms regarding the direct role of PCSK9 on the pathology of NAFLD.

Most recently, metabolic dysfunction-associated fatty liver disease (MAFLD) has been conceptualized to show a higher CVD risk than NAFLD because MAFLD has a basis of metabolic dysfunction (i.e., obesity, diabetes mellitus, and/or insulin resistance) [2,35,36]. Research on MAFLD will be required in the near future, as metabolic dysfunction was not fully investigated in the FH patients recruited in this study.

This study had certain limitations. The number of patients included in this study was comparatively small. This study was based on a cross-sectional design. It did not completely determine causality. This study focused on patients undergoing statin treatment. The data before statin administration were not examined. No pathological findings in the livers, for instance the imaging findings using the ultrasound technology that measures liver stiffness and/or histological findings by biopsy, were examined. The presence of hepatic dysfunction by statins could not be completely examined, while, at least, the test of hepatic function did not show extreme abnormality in this study population. Lifestyle habits, including diets that can be related to NAFLD, were not fully examined. The CVD events were not followed. These issues should be addressed in future works.

## 5. Conclusions

In this study, we found there to be a negative correlation between the PCSK9 levels and ALT:AST ratio, a marker of NAFLD risk, in patients with FH “under” statin treatment. Their negative correlation might give a hypothetical insight into the effect of statin treatment on the development of NAFLD, in relation to PCSK9 behavior, in this population.

## Figures and Tables

**Table 1:** Clinical characteristics of patients with FH under statin treatment.

Variables	Mean ± SD or Median (IQR)
Age, years	57 ± 10
Male, %	12%
BMI, kg/m^2^	23.2 ± 3.1
TG, mmol/L	1.50 ± 0.77
HDL-C, mmol/L	1.82 ± 0.53
LDL-C, mmol/L	3.38 ± 0.78
PCSK9, ng/mL	312 (263–344)
AST:ALT ratio	0.88 (0.77–1.07)

FH, familial hypercholesterolemia; SD, standard deviation; IQR, interquartile range; BMI, body mass index; TGs, triglycerides; HDL-C, high-density lipoprotein cholesterol; LDL-C, low-density lipoprotein cholesterol; PCSK9, proprotein convertase subtilisin kexin 9; ALT, alanine aminotransferase; AST, aspartate aminotransferase.

**Table 2:** Correlation with PCSK9 in patients with FH under statin treatment.

Variables	*r*	*p*-Value	*β*	*p*-Value
Age, years	−0.12	0.56	-	-
Male	−0.36	0.07	-	-
BMI, kg/m^2^	0.02	0.93	-	-
TG, mmol/L	−0.17	0.40	-	-
HDL-C, mmol/L	0.30	0.14	-	-
LDL-C, mmol/L	−0.36	0.07	−0.47	0.06
ALT:AST ratio	−0.45 *	0.02	−0.67 *	0.02

FH, familial hypercholesterolemia; BMI, body mass index; TG, triglyceride; HDL-C, high-density lipoprotein cholesterol; LDL-C, low-density lipoprotein cholesterol; PCSK9, proprotein convertase subtilisin kexin 9; ALT, alanine aminotransferase; AST, aspartate aminotransferase; * significant (correlation coefficient) < 0.05.

## Data Availability

Data will be made available upon reasonable and ethically proper requests from the corresponding author.
